# The Use of Electrocardiogram Smartwatches in Patients with Cardiac Implantable Electrical Devices

**DOI:** 10.3390/s24020527

**Published:** 2024-01-15

**Authors:** Marc Strik, Sylvain Ploux, Joske van der Zande, Anouk Velraeds, Leslie Fontagne, Michel Haïssaguerre, Pierre Bordachar

**Affiliations:** 1 Cardio-Thoracic Unit, Bordeaux University Hospital (CHU), F-33600 Pessac-Bordeaux, France; sylvain.ploux@gmail.com (S.P.); michel.haissaguerre@chu-bordeaux.fr (M.H.); bordacharp@hotmail.com (P.B.); 2 IHU Liryc, Electrophysiology and Heart Modeling Institute, Fondation Bordeaux Université, F-33600 Pessac-Bordeaux, France; joske.van.der.zande@gmail.com (J.v.d.Z.); anoukvelraeds@gmail.com (A.V.); 3 Cardiovascular and Respiratory Physiology, Twente University, 7522 NB Enschede, The Netherlands

**Keywords:** smartwatch, pacemaker, implantable cardioverter defibrillator, CIED, electrocardiogram

## Abstract

Unlimited access to ECGs using an over-the-counter smartwatch constitutes a real revolution for our discipline, and the application is rapidly expanding to include patients with cardiac implantable electronic devices (CIEDs) such as pacemakers (PMs) and implantable cardioverter defibrillators (ICDs). CIEDs require periodic evaluation and adjustment by healthcare professionals. In addition, implanted patients often present with symptoms that may be related to their PMs or ICDs. An ECG smartwatch could reveal information about device functioning, confirm normal device function, or aid in the case of device troubleshooting. In this review, we delve into the available evidence surrounding smartwatches with ECG registration and their integration into the care of patients with implanted pacemakers and ICDs. We explore safety considerations and the benefits and limitations associated with these wearables, drawing on relevant studies and case series from our own experience. By analyzing the current landscape of this emerging technology, we aim to provide a comprehensive overview that facilitates informed decision-making for both healthcare professionals and patients.

## 1. Introduction

Advancements in wearable technology have led to the development of smartwatches capable of monitoring various aspects of our health. These innovative devices not only provide timekeeping and fitness-tracking features but also offer the ability to register electrocardiograms (ECGs) [1]. By integrating ECG registration into smartwatches, users can conveniently capture and record their hearts’ electrical activity anytime and anywhere. Currently, smartwatches only screen ECGs for the presence of atrial fibrillation (AF), but an ECG can be evaluated for the presence of other abnormalities, and automatic diagnoses will likely be expanded in the near future [2]. An ECG smartwatch functions as a single-lead ECG recording device with one electrode on the back of the watch in contact with the wrist. The other electrode is on the front or side of the watch, and in order to register the ECG, the user needs to activate the recording and press the opposite index finger (or hand) on the watch crown (or watch surface). The list of smartwatches that include ECG capabilities is continuously growing. Most of the literature is based on a single model, but more smartwatches are becoming available that are less expensive and more compatible. Smartwatch ECGs empower patients to proactively monitor their heart health and detect potential abnormalities or irregularities promptly [3,4]. Moreover, with the ability to store and share ECG data, patients can actively participate in their healthcare journey by providing accurate information to healthcare providers, leading to more informed decision-making and personalized treatment plans [5]. Unlimited access to ECGs using an over-the-counter smartwatch constitutes a real revolution for our discipline, and the application is rapidly expanding to include patients with cardiac implantable electronic devices (CIEDs) such as pacemakers (PMs) and implantable cardioverter defibrillators (ICDs). CIEDs are essential tools for managing and treating cardiac conduction and rhythm disease. These devices work by monitoring the heart’s electrical activity and delivering therapeutic interventions when necessary. However, despite their life-saving benefits, they require periodic evaluation and adjustment by healthcare professionals [6]. In addition, patients implanted with a pacemaker or ICD often present with symptoms that may be related to their PMs or ICDs. An ECG smartwatch could reveal information about device functioning, confirm normal device function, or aid in the case of device troubleshooting. The advent of smartwatches equipped with ECG registration capabilities brings about the possibility of enhancing patient engagement and providing valuable insights into the cardiac function of individuals with implanted devices. However, the compatibility of smartwatches with implanted CIEDs is a crucial consideration. Concerns regarding electromagnetic interference and potential device malfunction exist, but few studies have examined the impact of smartwatches on implanted cardiac devices.

In this review, we delve into the available evidence surrounding smartwatches with ECG registration and their integration into the care of patients with implanted pacemakers and ICDs. We explore safety considerations and the benefits and limitations associated with these wearables, drawing on relevant studies and case series from our department. By analyzing the current landscape of this emerging technology, we aim to provide a comprehensive overview that facilitates informed decision-making for both healthcare professionals and patients.

## 2. Methods

### 2.1. Search Strategy

A systematic search was performed using PubMed in September 2023. A combination of MeSH terms and keywords related to this review (i.e., smartwatch or smart watch, ECG or electrocardiogram) were used with Boolean operators.

### 2.2. Study Selection

We followed the Preferred Reporting Items for Systematic Reviews and Meta-Analyses guidelines [7]. The initial search yielded 245 studies. Included studies: (1) were published in English, (2) focused on the use of smartwatches, and (3) focused on patients with pacemakers and ICDs. Fifteen duplicates were removed. After screening the abstracts, 95 articles were retrieved for a full-text review. A pair of reviewers independently reviewed the assigned articles in the first round; in the second round, all reviewers checked discrepancies and discussed inclusion/exclusion until agreement was reached. Finally, 29 articles were selected for review.

## 3. Safety and Precautions for Use

In theory, a smartwatch may influence CIEDs in multiple ways: by emission of electromagnetic interference (EMI), bioimpedance energy, or magnetic current. CIEDs are known to be susceptible to electromagnetic interference (EMI) as the oversensing of nonphysiologic signals may lead to inappropriate inhibition of pacing, inappropriate pacing during a vulnerable period, and inappropriate therapies. The risk of interaction between CIEDs and consumer devices became an area of interest in the 1990s with the emergence of the cell phone [8]. The largest study was performed by Hayes et al., who investigated EMI in 980 patients with CIEDs [9]. In total, 5625 tests were carried out using five different cell phones and during various situations. The authors reported clinically significant EMI of 6.6%, with EMI only occurring when the cell phone was held directly over the pacemaker. There were no cases of clinically significant EMI occurring when it was held at the normal position over the ear. Their study and other similar studies led to the widely adopted FDA recommendation that cell phones should be maintained at a distance of six inches (15 cm) away from CIEDs [10]. Newer generations of cell phones use other forms of communication systems that have different signal properties and lower maximum power emission, which have lower potential for CIED interference. Burri et al. reported no evidence of interference with smartphones during 882 tests in 63 ICD patients [11]. A study by Lacour et al. evaluated the incidence and consequences of electromagnetic interference produced by a smartwatch in patients with transvenous CIEDs and reported no evidence of device–device interaction [12]. Tzeis et al. performed 684 EMI tests in 171 CIED patients (71% pacemakers, 29% ICDs) using two smartwatch models [13]. Despite testing in the “worst-case” conditions with placement of the wearable directly above the implanted devices, the smartwatches did not cause any EMI in either study, neither with the implanted device nor the interrogation telemetry. Smartwatches have also been shown to be safer compared with other portable electronic devices such as electronic pens or in-ear headphones regarding the CIED interaction [14]. The very low energy emission by the smartwatches and the peak magnetic flux density emitted by the smartwatches, which was similar to the background noise level (0.81 μT), coupled with the technological advances in CIED platforms to filter and process the signals, explain this negligible to no risk of EMI [13]. More recently, a benchmark study evaluated electrical interference of bioimpedance technology available in selected smartwatches on CIEDs following the FDA’s accepted ISO 14177 standard [15]. Medical bioimpedance technology consists of applying an alternating, low-amplitude, painless electrical current via two electrodes and then measuring the resulting voltage generated by the body using a different pair of electrodes. Changes that occur as a result of disease will alter the ionic and cellular integrity of tissues and fluids, thus affecting their ability to conduct the electrical current, which will result in variations in the measurement. The technology can be used to detect edema in the lungs and the limbs, for example [16]. Simulations showed evidence of interference with voltage values exceeding threshold values. However, the ISO 14117 standard does not currently incorporate testing methodology or requirements for conducted current applied to the skin [17]. In fact, there is currently no existing standard that provides testing guidelines in this regard. Therefore, we can solely rely on clinical reports, of which there are none to date. CIEDs are also susceptible to magnets, as a magnetic field may temporarily deactivate ICD therapies and trigger asynchronous pacing in pacemakers. Recently, Greenberg et al. reported that the iPhone 12 series, which has a circular array of magnets around a central charging coil, suspended ICD therapies once an iPhone was brought close to an ICD over the left chest area [18]. At the same time, Nadeem et al. also reported inhibition of ICD therapies in three implanted patients when an iPhone was placed directly on the skin over the pocket [19]. The therapy suspension was immediate and persisted for the duration of the test. The magnets aid in properly aligning the smartphone on a wireless charger and other peripheral accessories and are also implemented within the newer iPhone models 13, 14, and 15. After these reports were published, the iPhone manufacturer (Apple) released a statement detailing precautionary measures for iPhone users with a cardiac implantable electronic device (CIED) in place. Recommendations were also made to keep the iPhone at a distance of ≥6 inches (15 cm) from the CIED as a safety measure to prevent the inhibition of lifesaving therapies [20]. Patel et al. repeated the experiment in 17 ICD patients, but to emulate a real-world scenario, the iPhone 12 was not placed directly over the skin above the device generator but instead was positioned over the patients’ clothes [21]. None of the device interrogations showed interruption of device therapies due to the iPhone. They concluded that, despite the iPhone having shown in vitro interference of ICD functioning, its effects are not clinically relevant in vivo. The precise distance of the phone from the device, the thickness of the clothing, and the thickness of subcutaneous tissue over the generator are some of the factors that could have a significant impact on the conflicting results. While the possibility of interference between a CIED and a smartphone is often well-known to implanted patients, the risk of interference between a smartwatch and a CIED is less well-known and is the subject of many questions during follow-up consultations. There have been a few studies that investigate a possible interaction between smartwatches and CIEDs. Small potent magnets such as those used in certain smartwatch wristband magnets have been reported to deactivate an ICD at distances of up to 2 cm [22,23]. Currently, there are no recommendations from CIED manufacturers regarding the use of smartwatches. In contrast, the manuals for the various smartwatches indicate that use is not advised in patients with a CIED. It is therefore relatively common to be questioned in our daily practice about the safety of using a smartwatch in patients implanted with a CIED. The lack of clinical adverse reports in the literature and in our own department led us to conclude that smartwatches can be safely used in patients with CIEDs as long as the devices are not placed in very close vicinity to each other.

## 4. Automatic Diagnosis of AF

While the diagnosis of AF is the only automatic diagnosis proposed by smartwatches today, the clinical interest in patients implanted with a CIED is very modest as the diagnosis is readily made by interrogation of the device or with remote monitoring. The smartwatch may diagnose AF in two ways: with an irregular rhythm alert (based on continuous photoplethysmography) or with an irregular rhythm as measured on the ECG [24,25]. Table 1 shows the various ECG smartwatches available on the market with the most important differences between the models. The diagnostic accuracy of the continuous photoplethysmography approach using a smartwatch to detect episodes of AF was investigated in thirty participants with known paroxysmal AF implanted with a CIED. In eleven subjects who had AF as registered by their CIED while a smartwatch was worn, eight were alerted with an irregular rhythm alert [26]. There were a total of 70 AF episodes registered by the CIED, 35 of which occurred while the smartwatch was being worn. Of these, 21 were detected with an irregular rhythm alert with one false positive. In patients who do not have remote monitoring, the use of a smartwatch has been reported to provide an earlier diagnosis of AF [27]. Also, in the absence of irregular rhythm alerts, a patient implanted with a CIED may self-register an ECG with a smartwatch and get confused by the automatic diagnosis stating the presence or absence of AF. The accuracy of the various smartwatches for the diagnosis of AF has been mostly validated in non-implanted patients. For all smartwatches on the market today, the diagnosis of AF is based on the irregularity of the QRS complexes and is therefore unsuitable in implanted patients who have atrioventricular block (risk of false negative result) or premature complexes (risk of false positive result). We previously assessed the diagnostic accuracy of a smartwatch automated diagnosis of AF in a large population of hospitalized adult patients with various ECG anomalies [28]. In a total of 734 patients, 39 (5.3%) had a ventricular-paced rhythm. Compared with patients without a PM, the risk of having false positive tracings (false diagnosis of AF) was not significantly higher for patients with pacing (RR 1.72, 95% CI 0.94–3.15, *p* = 0.08). In contrast, as expected, the risk of having false negative tracings (missed AF) was higher for patients with pacing (RR 2.47, 95% CI 1.19–5.03; *p* = 0.02). Compared with patients without the abnormality, the risk of having false positive tracings was higher for patients with PACs/PVCs (RR 2.9, 95% CI 1.9–4.4, *p* < 0.0001). It is therefore important to inform the implanted patient about the risk of misdiagnosis when using an ECG smartwatch. Guidance for the use of smartwatches for detecting AF in implanted or non-implanted patients remains scarce, and widespread clinical application and experience are the most likely sources for definite answers [29].

## 5. Diagnosis of Palpitations or Pre-Syncope

**Figure 1:**
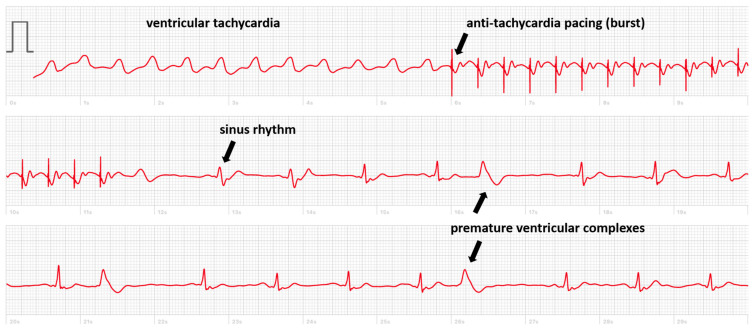
Smartwatch ECG showing a ventricular tachycardia treated by anti-tachycardia pacing (burst) with the re-appearance of sinus rhythm with premature ventricular complexes.

The interest in smartwatch ECGs for non-implanted patients who have palpitations of pre-syncope seems clear as the diagnosis of arrhythmia (or lack thereof) can be made on demand [2,35]. Interestingly, smartwatches may prevent unnecessary CIED implantation, as shown by Liu et al., who sent 100 patients home with a smartwatch after transcatheter valve implantation of whom four were remotely identified as requiring pacemaker implantation [36,37]. But also, patients implanted with a CIED may present with symptoms of palpitations of pre-syncope without diagnosis provided by the memories of the PM or ICD due to the absence of recording an EGM or an algorithm dysfunction. In the literature, we found a case of a 32-year-old male implanted with a dual-chamber pacemaker for atrioventricular block presenting with exercise intolerance. The diagnosis of a 2/1 effort block was made by his smartwatch, which showed a sudden drop in heart rate from 150 to 75 bpm using the photoplethysmography sensor [38]. In our department, we sometimes evaluate symptomatic implanted patients who have recorded an ECG with their smartwatch. In one case, a patient implanted with an ICD recorded an ECG during palpitations showing ventricular tachycardia terminated by anti-tachycardia pacing (Figure 1). Of course, in these situations, the device will have registered the tachycardia simultaneously, and the added value provided by the smartwatch is limited. Identifying whether the QRS complexes during the tachycardia are positive or negative in lead I may be informative for a possible ablation procedure.

**Figure 2:**
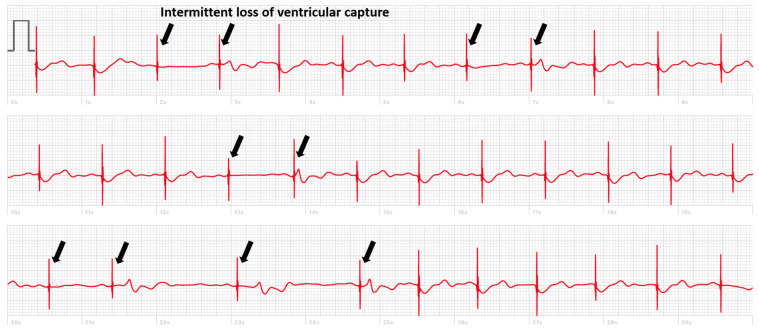
Smartwatch ECG of a patient implanted with a pacemaker, arrows indicate intermittent loss of ventricular capture.

**Figure 3:**
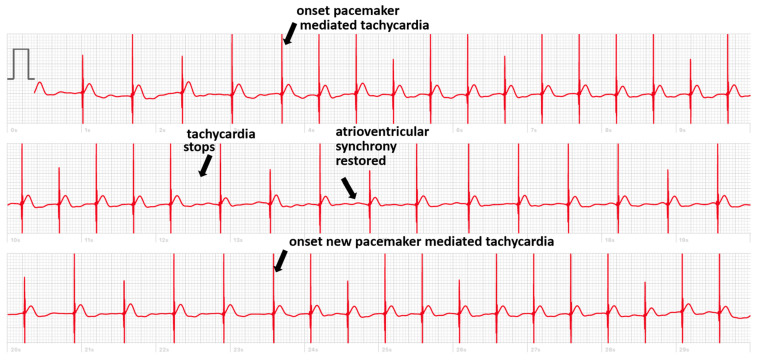
Smartwatch ECG showing ventricular pacing with two episodes of pacemaker-mediated tachycardia.

But there are also cases where, in the absence of simultaneous recording with the implanted device, the smartwatch can deliver a diagnosis. We present the case of a patient implanted with a dual chamber PM who registered a smartwatch ECG made during symptoms of pre-syncope showing an intermittent loss of capture (Figure 2). In another case, the smartwatch ECG revealed the occurrence of pacemaker-mediated tachycardia (PMT) in a patient implanted with a Medtronic pacemaker presenting with palpitations (Figure 3). These pacemakers do not record an EGM tracing for storage in their memory during an episode of PMT. The tracing showed electro-driven tachycardia at the ventricular stage at the maximum tracking rate (130 bpm). This tracing also showed the difficulty of interpreting a single-lead ECG tracing in paced patients. Several possibilities were possible in addition to PMT such as atrial tachycardia and 2-to-1 atrial flutter. Careful interrogation of the pacemaker revealed intermittent loss of atrial capture with the recurrence of PMT, confirming the diagnosis suspected based on the smartwatch ECG.

## 6. Smartwatches in Patients Implanted with a Leadless Pacemaker

**Figure 4:**
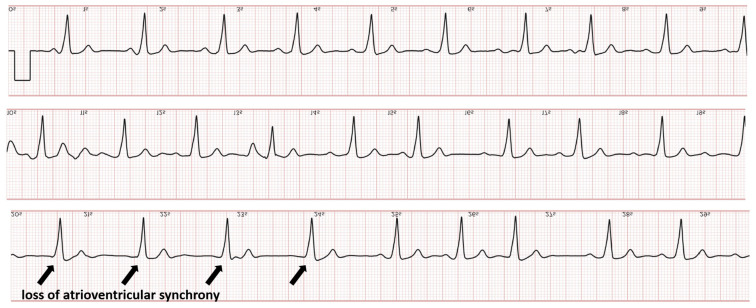
Smartwatch ECG showing ventricular pacing with proper atrioventricular synchronization at the onset but the loss of atrioventricular synchrony during the second and third tracing.

Leadless pacemakers offer an alternative to conventional transvenous pacing systems, particularly for patients at an increased risk of infection or with limited venous access [39]. The Micra (Medtronic) wireless pacemaker is mostly implanted in patients who do not need ventricular tracking of the atrial rhythm, for example, patients with permanent fibrillation. This is because the capsule is implanted in the ventricle and the device only senses events from the ventricle, not the atrium. To widen the spectrum of patients who qualify for leadless pacing, a second-generation version was developed providing atrio-ventricular synchrony (Micra AV) [40]. In the Micra AV, the accelerometer-derived movement waveform is used to sense the mechanical contribution of the right atrium. Ventricular pacing is timed to synchronize with atrial mechanical-sensed events. However, obtaining adequate AV synchrony with this novel technology is challenging, and extensive programming optimization is required to increase the proportion of patients in whom AV synchrony can be achieved [41]. Current leadless pacemakers do not automatically trigger EGM recordings during tachycardia or the loss of atrioventricular synchronization. We recently decided to dedicate a part of our remote monitoring resources to offer a smartwatch solution to patients with sporadic symptoms. As part of this project, we equipped all our patients implanted with a Micra AV with an ECG smartwatch with the instruction to record a routine tracing systematically every week (and also, in the case of symptoms) both in the standing and supine position. Despite programming optimization at predischarge, the success in achieving AV synchrony was intermediate in many patients, which required further adjustment of atrial sensing parameters. Smartwatch ECGs allowed for the early recognition of failure to detect atrial contraction; Figure 4 shows an example of intermittent atrial undersensing resulting in ventricular pacing at the lower rate of 50 beats per minute.

## 7. Using Smartwatches to Monitor Left Ventricular Capture

**Figure 5:**
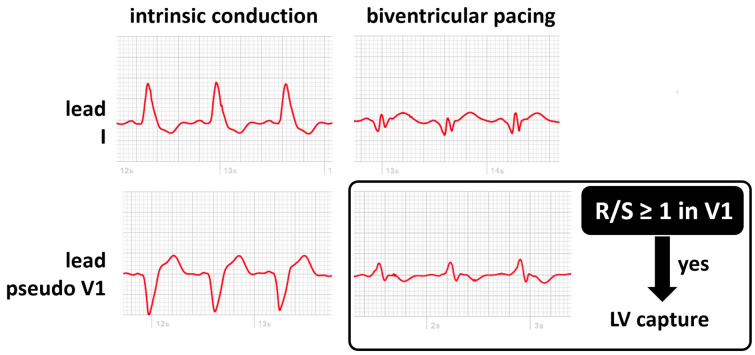
Smartwatch ECG during intrinsic conduction (absence of biventricular pacing, **left panels**) and during biventricular pacing (**right panels**) on the wrist (lead I, **top panels**) and on the chest (lead pseudo V1, **bottom panels**). LV capture can be confirmed if the R/S ratio is at least 1 in the V1 position.

The benefits described with cardiac resynchronization therapy (CRT) directly depend on the maintenance of continuous biventricular pacing. Loss of left ventricular (LV) capture may be difficult to diagnose without a programmer and often goes unnoticed between device clinic visits. An ECG-based algorithm using the ventricular polarity in leads I and V_1_ of the standard 12-lead electrocardiogram has demonstrated high accuracy in detecting loss of left ventricular capture [42]. Monitoring LV capture may represent another application of smartwatches in CRT patients. A recent study investigated the use of applying a modified Amman algorithm on two single-lead ECGs acquired with a smartwatch: one on the wrist (lead I) and one on the chest (V1 position) [43]. They reported that a smartwatch diagnosed loss of LV capture in patients with a similar accuracy compared to previous reports using a standard 12-lead ECG. When comparing smartwatch ECGs during biventricular pacing and right ventricular pacing, loss of LV pacing was correctly identified in 25 of 28 patients (89%). Based on the results of this cited study, smartwatches are routinely used in our center in patients with known high left ventricular thresholds to screen for loss of LV capture. Before discharge, lead I and modified lead V_1_ ECGs are recorded using a smartwatch in spontaneous rhythm, RV, LV, and BV pacing. Once at home, the patient records and transmits periodically lead I and modified lead V_1_, allowing for the monitoring of efficient LV capture (Figure 5).

## 8. Smartwatches in Patients with Left Bundle Branch Area Pacing

**Figure 6:**
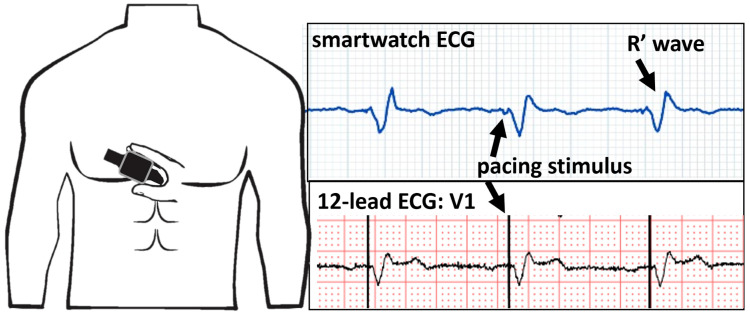
Smartwatch ECG on the V1 position (**top panel**) and the V1 lead of the 12-lead ECG (**bottom panel**) showing unipolar ventricular pacing with the characteristic R′ wave signifying capture of the left bundle branch.

Left bundle branch area pacing is an emerging alternative to biventricular pacing in the quest for optimal and physiological pacing. The 12-lead ECG allows for the discrimination between selective, non-selective-left bundle branch pacing, and left ventricular septal pacing. One of the major concerns of this new technology is safety issues such as delayed increases in capture thresholds. As for CRT, smartwatches have been proposed to monitor the maintenance of efficient capture with left bundle branch area pacing. A case series performed on patients with His bundle pacing and left bundle branch area pacing documented technical issues with a certain smartwatch model and the failure to record ECGs when the pacing configuration was unipolar. When the pacing polarity was programmed to bipolar, the smartwatch was able to correctly register an ECG. In our center, we use another smartwatch model that does not have this issue, and placing the watch on the V1 chest position reveals the R prime wave typical for left bundle area capture (Figure 6).

## 9. Opportunities for CIED Remote Follow-Up

More and more patients perform manual or automated transmissions of their CIED at home, which enables the possibility of performing remote device check-ups in the form of teleconsultations. However, the surface electrocardiogram remains an essential investigation during a routine device clinical follow-up as it may reveal issues of undersensing or oversensing that are missed by the CIED. This may prove particularly useful in the setting where an ECG is required to enable reimbursement of the remote CIED check-up. Here, the ECG smartwatch may also benefit the patient as it enables the registering of an ECG at home in preparation for the teleconsultation. A smartwatch ECG may be limited when compared to an in-clinic twelve-lead ECG by the fact that it is a single derivation solution. However, the tracings are thirty seconds long, meaning there are three times more tracings than a conventional ECG. During CIED follow-up, anomalies visible on the ECG are most often paroxysmal and risk not showing up on a 10 s tracing. Specific use cases exist where the smartwatch ECG can be particularly interesting:

- Leadless pacemaker with atrial tracking (Micra AV, Medtronic or Aveir DR, Abbott): while the device interrogation reports the amount of ventricular pacing that is synchronized to the atrium, this figure may be incorrect and needs ECG verification. This holds true certainly in cases where atrioventricular synchrony is suboptimal.
- New pacing technologies such as left bundle branch area pacing are used more and more, but it is not clear whether automatic algorithms such as auto-threshold are reliable. The recording of a smartwatch ECG confirms capture.
- Single-chamber ICDs and subcutaneous ICDs now register an EGM when there is suspicion of atrial fibrillation. At the moment, these algorithms are only based on irregularity, which makes them unreliable with the occurrence of many false positives. The registration of the cardiac rhythm using a smartwatch ECG can aid in rejecting the diagnosis, avoiding unnecessary in-clinic investigation and inappropriate treatment (e.g., anticoagulation).

## 10. Smartwatch Applications beyond the ECG

While the possibility of registering an ECG holds many opportunities in patients implanted with a PM or ICD, the smartwatch boasts many other features that can improve follow-up significantly. Smartwatches can be used by patients implanted with a pacemaker in several ways to help monitor their health and stay informed about their cardiac condition. However, it is important to note that the specific functionality may vary depending on the smartwatch model and the accompanying mobile device. The following are some ways smartwatches can be used:

- Heart rate monitoring: Most smartwatches come equipped with heart rate sensors that continuously monitor the heart rate using photoplethysmography. This can be useful for patients with pacemakers to ensure that their heart rate is within the desired range set by their healthcare provider. This is especially true in the case of patients with active rate modulation, in whom it is often difficult to find the correct settings for sensor sensitivity and rate-responsive aggressiveness. It is possible to obtain reliable heart rate curves with the use of photoplethysmography. Displayed heart rate at peak activity or even increases and decreases in the heart rate can provide very helpful information. Heart rate notifications: Some smartwatches can notify users if their heart rate goes above or below a certain threshold. This feature can be configured to alert patients if their heart rate exceeds safe limits, allowing them to seek medical attention if necessary.
- Activity tracking: Smartwatches can track daily activity levels, including steps taken, distance walked, and calories burned. Most PM and ICDs also have activity trackers based on the activation of the sensor, but this information is not readily available to the patient. Patients can use this data to monitor their overall physical health and ensure they are staying active within the limits recommended by their healthcare provider.
- Fall detection and emergency alerts: Many smartwatches include fall detection features that can automatically alert emergency services or a designated contact in case of a fall. This can be especially important for elderly patients with pacemakers who may be at a higher risk of falls.
- Medication and appointment reminders: Smartwatches can be used to set reminders for taking medications and attending medical appointments. This can help patients with pacemakers and ICDs adhere to their treatment plans.
- Sleep tracking: Monitoring sleep patterns can be important for overall health. Some smartwatches offer sleep-tracking features that can provide insights into sleep quality, which can indirectly impact cardiac health.

Combined with the remote monitoring capabilities of modern pacemakers, the concomitant use of a smartwatch may empower device patient follow-up in a synergistic manner. To effectively use a smartwatch in conjunction with a pacemaker or ICD, patients should consult with their healthcare provider to ensure compatibility and receive guidance on setting up and using the device. Additionally, it is important to remember that a smartwatch is not a replacement for regular check-ups and consultations with a healthcare professional, especially for patients with pacemakers who require ongoing medical management.

## 11. Future Directions

Follow-up of PM and ICD patients is increasingly performed remotely and as the need for a high-quality ECG persists, the use of the ECG smartwatch fits perfectly with this evolution. While current existing experience is limited, the available data show that ECG smartwatches may aid in PM/ICD follow-up as they may confirm proper device functioning or reveal inappropriate device behavior. More data on the structured use of ECG smartwatches in patients implanted with CIEDs is needed to learn more about patient acceptance, diagnostic yield, burden on healthcare professionals, and associated costs [44]. There is a need for large clinical studies that validate the detection of a wide range of arrhythmias that may be responsible for palpitations in patients with a PM/ICD. Although most consumer ECG devices today provide high-quality signals and, with the right algorithm, may be able to detect a wide spectrum of arrhythmias, many of the devices have only been validated for AF detection. Further algorithm developments will increase their accuracy and reliability and improve the clinical use of smartwatch ECGs.

## 12. Conclusions

The integration of smartwatches with ECG registration capabilities into the management of patients with implanted pacemakers and ICDs represents a significant advancement in the field of cardiac care. The evidence reviewed in this manuscript underscores the potential of smartwatches in enhancing patient engagement, providing valuable diagnostic insights, and enforcing remote monitoring. The safety considerations, specifically concerning electromagnetic interference and potential device malfunction, have been meticulously explored. Studies have demonstrated that smartwatches pose minimal risk to implanted devices when used at appropriate distances. ECG smartwatches hold immense potential in the diagnosis and management of various CIED challenges, including pacemaker-mediated tachycardia, monitoring ventricular capture, and evaluating the efficiency of new pacing technologies like left bundle branch area pacing. While challenges such as the misdiagnosis of atrial fibrillation and limitations in specific pacing technologies exist, ongoing research and algorithm developments hold promise for addressing these concerns. Looking ahead, the integration of ECG smartwatches into cardiac care is poised for further growth and refinement. Large-scale clinical studies are necessary to validate the accuracy and reliability of smartwatch ECGs for detecting a wide array of CIED challenges. As these devices continue to evolve, they hold the potential to revolutionize patient care by providing real-time, comprehensive, and personalized cardiac monitoring.

## Figures and Tables

**Table 1:** Comparison of 6 smartwatches with ECG capability. Price (USD), sensitivity/specificity for automatic AF diagnosis, heart rate range for automatic interpretation, battery life, water resistance (IP rating), and health features.

Aspect	Model 1	Model 2	Model 3	Model 4	Model 5	Model 6
**Price range**	USD 399–USD 799 [30]	USD 249–USD 329 [31]	USD 199–USD 249 [32]	USD 349 [33]	USD 349 [32]	USD 299–USD 499 [34]
**Sensitivity/specificity for diagnosing AF** [24,25]	85–87%/75–86%	85–88%/75–81%	66%/79%	Not available	Not available	58–78%/75–80%
**Limit of heart rate for interpretation**	50–150 beats/min	50–120 beats/min	50–120 beats/min	50–120 beats/min	50–120 beats/min	50–100 beats/min
**Battery life**	18 h	45 h	144 h	9 days	24 h	30 days
**Water resistance**	50 m–100 m	50 m	50 m	50 m	50 m	50 m
**Health features**	ECG, SpO2, fitness rings, fall detection	ECG, SpO2, stress tracking	ECG, SpO2, stress tracking	ECG, SpO2, body energy	ECG, SpO2, stress tracking	ECG, SpO2, breathing, temperature

## Data Availability

Not applicable.
